# tension: A Python package for FORCE learning

**DOI:** 10.1371/journal.pcbi.1010722

**Published:** 2022-12-19

**Authors:** Lu Bin Liu, Attila Losonczy, Zhenrui Liao

**Affiliations:** 1 Columbia University, New York, New York, United States of America; 2 Zuckerman Mind Brain and Behavior Institute, Columbia University, New York, New York, United States of America; 3 Department of Neuroscience, Columbia University, New York, New York, United States of America; Ghent University, BELGIUM

## Abstract

First-Order, Reduced and Controlled Error (FORCE) learning and its variants are widely used to train chaotic recurrent neural networks (RNNs), and outperform gradient methods on certain tasks. However, there is currently no standard software framework for FORCE learning. We present tension, an object-oriented, open-source Python package that implements a TensorFlow / Keras API for FORCE. We show how rate networks, spiking networks, and networks constrained by biological data can all be trained using a shared, easily extensible high-level API. With the same resources, our implementation outperforms a conventional RNN in loss and published FORCE implementations in runtime. Our work here makes FORCE training chaotic RNNs accessible and simple to iterate, and facilitates modeling of how behaviors of interest emerge from neural dynamics.

This is a *PLOS Computational Biology* Software paper.

## 1 Introduction

Recurrent neural networks (RNNs) are powerful tools for solving sequential tasks, from time-series modeling [1, 2] to natural language processing [3, 4] to robotics [5, 6] to modeling the dynamics of biological neural networks. RNNs are commonly trained using backpropagation through time via stochastic gradient descent (SGD), though long-term dependencies remain a vexing problem: any delay from an input to a desired outcome makes it harder to correctly identify and update the weights needed to give rise to that outcome, due to the intervening (possibly chaotic) dynamics during the delay period [7, 8]. Prior to the dominance of gradient-based methods, reservoir computing approaches enjoyed popularity in training RNNs, and in certain temporal tasks empirically converge in fewer samples/epochs compared to SGD [9–11]. First-Order, Reduced and Controlled Error (FORCE) learning [12] uses a recursive least-squares (RLS) rule to train networks to compute with chaotic dynamics, by analogy with how brain areas such as motor cortex are thought to compute [13]. FORCE learning is widely used in computational neuroscience to train chaotic RNNs to perform biologically-inspired tasks with long delays between input and desired output, which have traditionally been difficult for gradient-based methods [11, 14–19]. One advantage of the FORCE approach is that the internal dynamics of the network can easily be constrained with experimentally-measured neural dynamics, opening an avenue for interrogating how observed neural dynamics may give rise to target behavior in the brain [12, 17, 20, 21].

There does not currently exist an out-of-the-box library for FORCE-training RNNs. Our literature survey found that papers using FORCE all used custom implementations [12, 15, 16, 22–24], duplicating work and limiting direct extensibility. Moreover, producing a custom FORCE implementation which is both correct and high-performance may pose an obstacle to use by non-specialists. Here, we present tension, a FORCE learning and reservoir computing library based on TensorFlow / Keras. tension provides an API for FORCE learning in a familiar high-level interface while also offering the well-developed performance optimization tools of TensorFlow. Our models can easily be incorporated into an existing TensorFlow training and evaluation workflow.

This brief report provides an overview of the tension package. In the next section, we briefly review the FORCE algorithm and theory and we describe the usage and capabilities of the package. Finally, we demonstrate the use of our package to train rate networks and spiking networks to generate intrinsic dynamics, to perform a delayed-response task, and to reproduce biological data. Source code for all examples is available online.

## 2 Design and implementation

### 2.1 Background and theory

**Fig 1:**
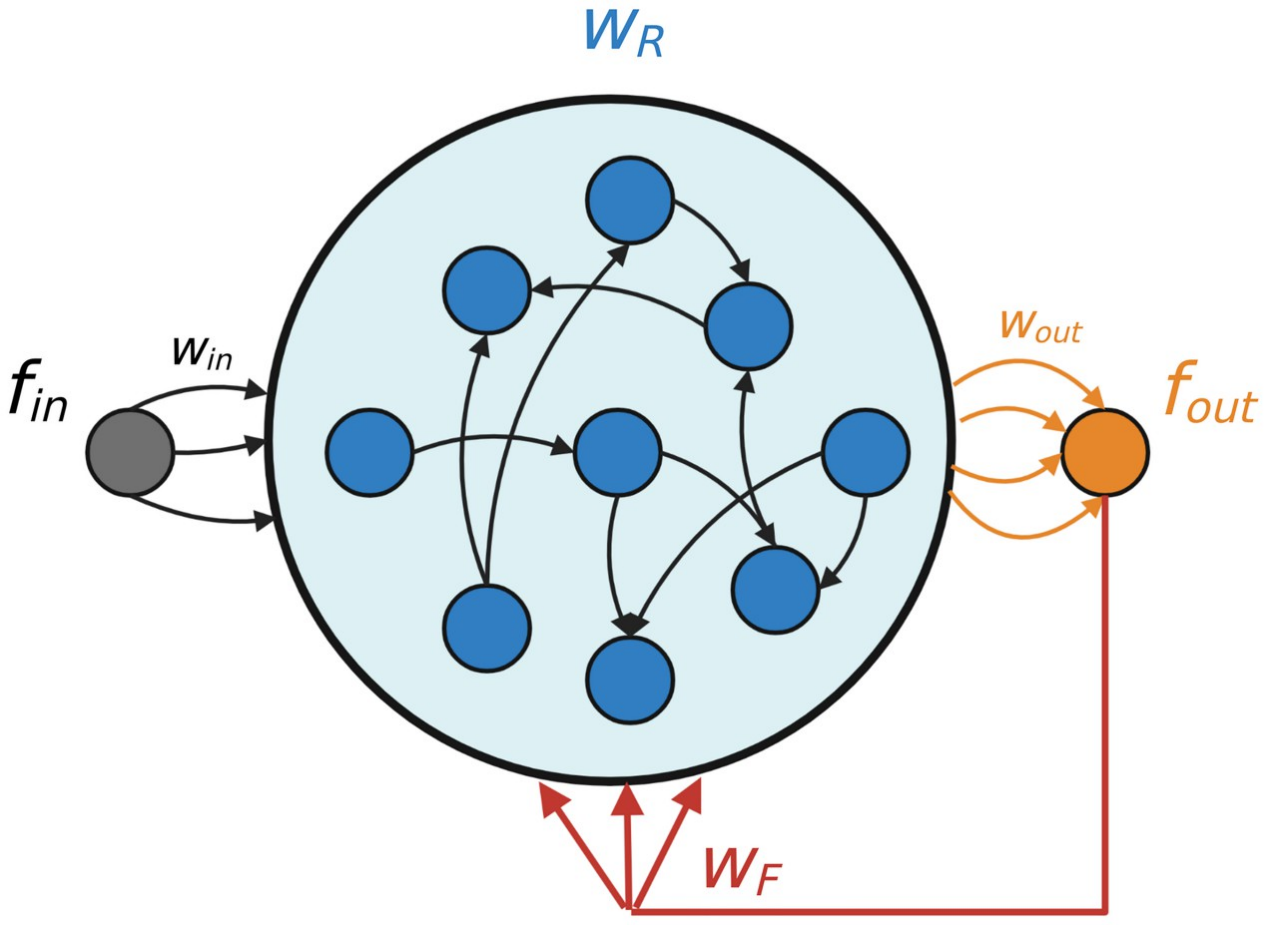
Illustration of a generic chaotic RNN Layer. The network receives inputs **f**_*in*_(*t*). Training modifies either **w**_*out*_ or **w**_*out*_ and **w**_*R*_ so that the network output *z*(*t*) matches a target *f*_*out*_(*t*).

In this section, we will mainly consider rate networks with the basic architecture of Fig 1 [10, 12] (though in Section A.3 in S1 Appendix we discuss a modification to this architecture that facilitates training; see [15]). We will refer to the most widely used recursive least-squares (RLS) algorithm for FORCE as simply the “FORCE algorithm”, though our framework is easy to extend to variants which do not depend on RLS. For a discussion of the theory of full-FORCE [15] and FORCE with spiking networks [16], which our package also supports, see the Section A in S1 Appendix.

We view the RNN as a dynamical system consisting of *N* neurons with currents **x**(*t*) and rates **r**(*t*) = *H*(*x*_*i*_(*t*)). There are four sets of (matrix-valued, in the general case) weights in the network: input weights **w**_*in*_, recurrent weights **w**_*R*_, output weights **w**_*out*_, and feedback weights **w**_*F*_. Learning in this network is defined as modifying the output and/or recurrent weights to reduce the error between a linear readout from the network $z\left(t\right)=w_{out}^{T}\left(t-\Delta t\right)r\left(t\right)$ and target function *f*_*out*_(*t*). Sometimes it will be convenient to only modify a subset of these weights, or to set some (e.g., **w**_*F*_, **w**_*in*_) equal to 0. The desired output may depend on a network input **f**_*in*_(*t*) or be entirely internally-generated.

**Algorithm 1**: The basic FORCE RLS algorithm

**Data**: RNN as in Fig 1, target output *f*_*out*_(*t*), (optional) input **f**_*in*_(*t*), timestep Δ*t*

**Result**: Trained output weights **w**_*out*_

*t* ← 0;

**w**_*out*_(0)← random initialization;

**P**(0) ← *α*^−1^**I**;   /* **I** is the identity matrix */

**while** *stopping criteria not met* **do**

 Update activations **x** using forward-pass (Eq 1);

 Apply activation function to generate rates **r**(*t*);

 Update current output *z*(*t*);

 Update **P**(*t*);

 Update error *e*_−_(*t*);

 Calculate pseudogradient **Δw**;

 Update weights;

**end**

FORCE learning improves upon previous reservoir methods for training the Echo-State Network (ESN) [10, 25] (also see Section A.6 in S1 Appendix), controlling the error between target and true output at every training timestep by feeding the true output back to the network during training [12]. The steps of the algorithm are summarized in Algorithm 1 (update equations in the Section A in S1 Appendix).

The discrete time forward pass, adapted from the continuous time differential equation common to the reservoir computing and FORCE frameworks [10, 12], is given by:

$$\begin{array}{cc} \tau \frac{\Delta x^{T}}{\Delta t} & =-x^{T}\left(t-\Delta t\right)+f_{in}^{T}\left(t\right)w_{in}+r^{T}\left(t-\Delta t\right)w_{R}\left(t-\Delta t\right)+z^{T}\left(t-\Delta t\right)w_{F} \end{array}$$ (1)

where **f**_*in*_(*t*) is a multi-dimensional input, **x**(*t*) the *N* dimensional pre-activation neuron firing rates, *τ* the time-constant, and *H*(⋅) some activation function.

Unlike the classic ESN, the *N* × *N* recurrent weight matrix **w**_*R*_ may also be trainable rather than static. If trainable, the update rules for **w**_*R*_ are given by [12] (assuming a scalar output):

$$\begin{array}{cc} A_{jk}^{i}\left(t\right) & =A_{jk}^{i}\left(t-\Delta t\right)-\frac{\sum_{l\in B(i)}\sum_{m\in B(i)}\;A_{jl}^{i}\left(t-\Delta t\right)r_{l}\left(t\right)r_{m}\left(t\right)A_{mk}^{i}\left(t-\Delta t\right)}{1+\sum_{l\in B(i)}\sum_{m\in B(i)}r_{l}\left(t\right)A_{lm}^{i}\left(t-\Delta t\right)r_{m}\left(t\right)}\\ w_{R}^{ji}\left(t\right) & =w_{R}^{ji}\left(t-\Delta t\right)-e_{-}\left(t\right)\sum_{k\in B(i)}A_{ik}^{j}\left(t\right)r_{k}\left(t\right) \end{array}$$

where *B*(*i*) are the set of neurons presynaptic to neuron *i*.

In the backward pass, an error *e*_−_(*t*) is computed between the target and a linear readout from the network. A delta-type learning rule is applied to update the readout weights

$$\begin{array}{c} w_{out}\left(t\right)=w_{out}\left(t-\Delta t\right)-e_{-}\left(t\right)P\left(t\right)r\left(t\right) \end{array}$$ (2)

where **P**(*t*) is a running estimate of the inverse regularized correlation matrix of the network rates **r**(*t*). For TensorFlow / Keras compatibility, we simply drop in the weight update −*e*_−_(*t*)**P**(*t*)**r**(*t*) as a “pseudogradient”, where **P**(*t*) can be thought of as a direction-dependent learning rate matrix [12].

**Fig 2:**
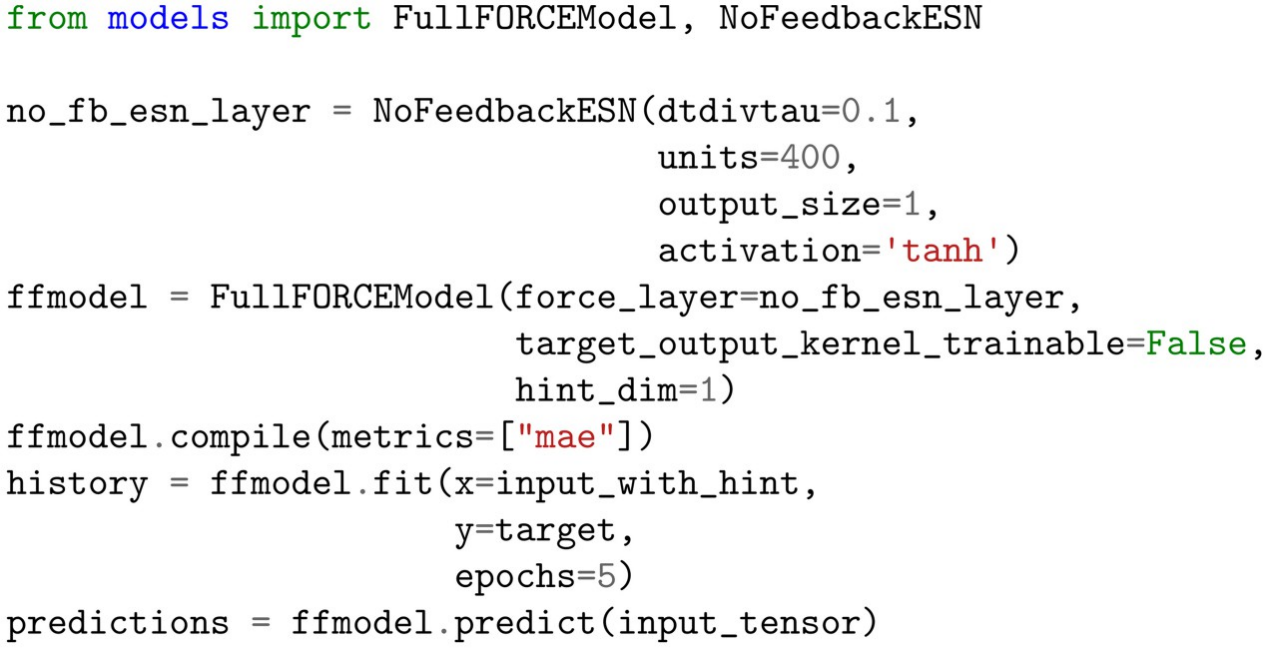
API for full-FORCE training of a chaotic RNN without feedback.

Our package is built on top of Tensorflow / Keras. In tension, FORCE models can be defined and fit in 5 lines of code as illustrated in the code snippet in Fig 2. At a high level, defining and training a model consists of the following steps (identical to the Keras interface):

1. Define a FORCELayer object (chaotic RNN)
2. Define a FORCEModel object
3. Compile the model by calling FORCEModel.compile(…)
4. Fit the model by calling FORCEModel.fit(…), along with any necessary callbacks
5. Get predictions from the model by calling FORCEModel.predict(…)

FORCELayer classes define the network internal dynamics independent of training. In the “forward pass”, the basic RNN architecture trained by FORCE is identical to the ESN. They differ in the actual training algorithm used, which is defined by the FORCEModel class. FORCEModel and FORCELayer classes can be independently recombined; thus our package can also be used as a general-purpose package for reservoir computing.

Base classes in our API are designed with Keras principles such as modularity and progressive disclosure of complexity in mind, with semantically atomic and reusable methods [26, 27]. Thus, users can easily define new Model and Layer objects for their custom applications and architectures by subclassing and overwriting the relevant base class methods.

### 2.2 API inheritance structure

**Fig 3:**
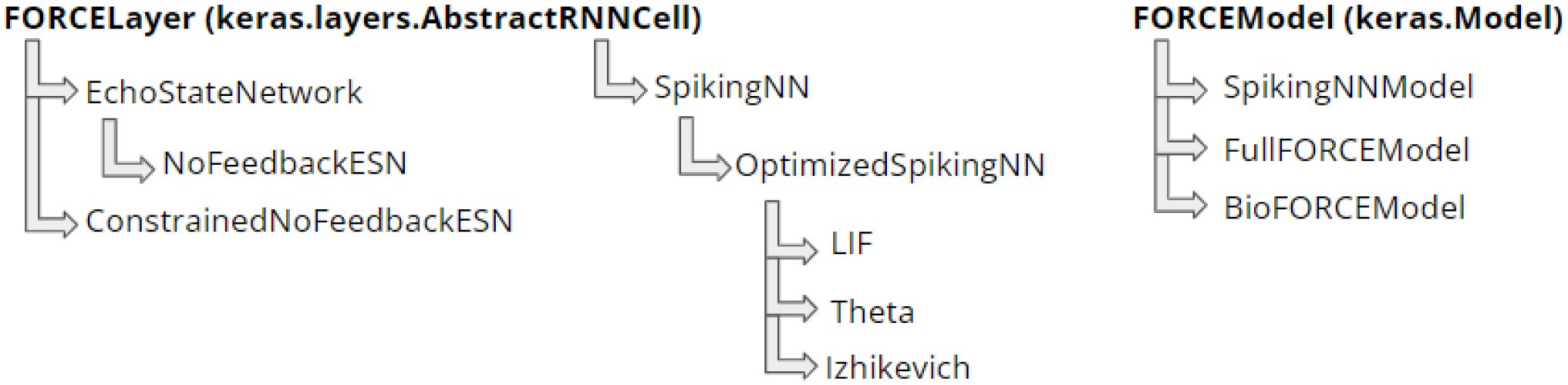
API inheritance structure.

We provide out-of-the-box implementations of commonly used RNN architectures as FORCE Layer classes which can be trained using different Model classes (Fig 3). The class definitions for these FORCE Layer classes are:

- FORCELayer(keras.layers.AbstractRNNCell): base chaotic RNN layer class defining the weight initialization.
- EchoStateNetwork(FORCELayer) and NoFeedbackESN(EchoStateNetwork): defines RNNs with and without feedback, respectively.
- ConstrainedNoFeedbackESN(FORCELayer): variant of NoFeedbackESN constrained by a structural connectome between neurons and has dynamics as outlined in [22].
- SpikingNN(FORCELayer) and OptimizedSpikingNN(SpikingNN): defines high level methods for building spiking chaotic RNN layers per [16].
- LIF(OptimizedSpikingNN), Izhikevich(OptimizedSpikingNN), and Theta(OptimizedSpikingNN): implementation of leaky-integrate and fire (LIF), Izhikevich, and theta spiking neural networks with forward pass as outlined in [16]. Section A.2 in S1 Appendix summarizes the voltage rate equations for these networks.

and the following FORCE model class definitions:

- FORCEModel(keras.Model): base FORCE model class that implements FORCE learning per [12]. The base model class is compatible with EchoStateNetwork and NoFeedbackESN.
- FullFORCEModel(FORCEModel): implements full-FORCE algorithm from [15] for training EchoStateNetwork and NoFeedbackESN.
- SpikingNNModel(FORCEModel): applies FORCE algorithm for training SpikingNN subclasses LIF, Izhikevich, and Theta.
- BioFORCEModel(FORCEModel): adapted based on [22] for FORCE training of ConstrainedNoFeedbackESN constrained by empirically recorded data.

### 2.3 Defining new rate network layers

Custom rate network layers can be created by sub-classing FORCELayer and defining the following methods:

- call(self, inputs, states) method defining the forward pass of the RNN. The method takes in the input at some timestep with the current states of the layer and returns the output of the forward pass with the new states of the layer.
- get_initial_states(self, inputs = None, batch_size = None, dtype = None) that returns the initial states of the layer during the initial layer call.

### 2.4 Defining new spiking neural network layers

Custom spiking neural network layers can be created by sub-classing SpikingNN or OptimizedSpikingNN and defining the following methods:

- initialize_voltage(self, batch_size) returns the initial voltage of each neuron in the spiking neural networks layer.
- update_voltage(self, I, states) returns the updated voltages of each neuron (plus two other tensors of the same size).

In addition to the required methods listed above, any pre-existing method in base classes may be modified (state size, kernel initialization etc.) via subclassing so long as the input and output adheres to the specifications indicated in the documentation.

## 3 Results

### 3.1 Basic performance evaluation

**Fig 4:**
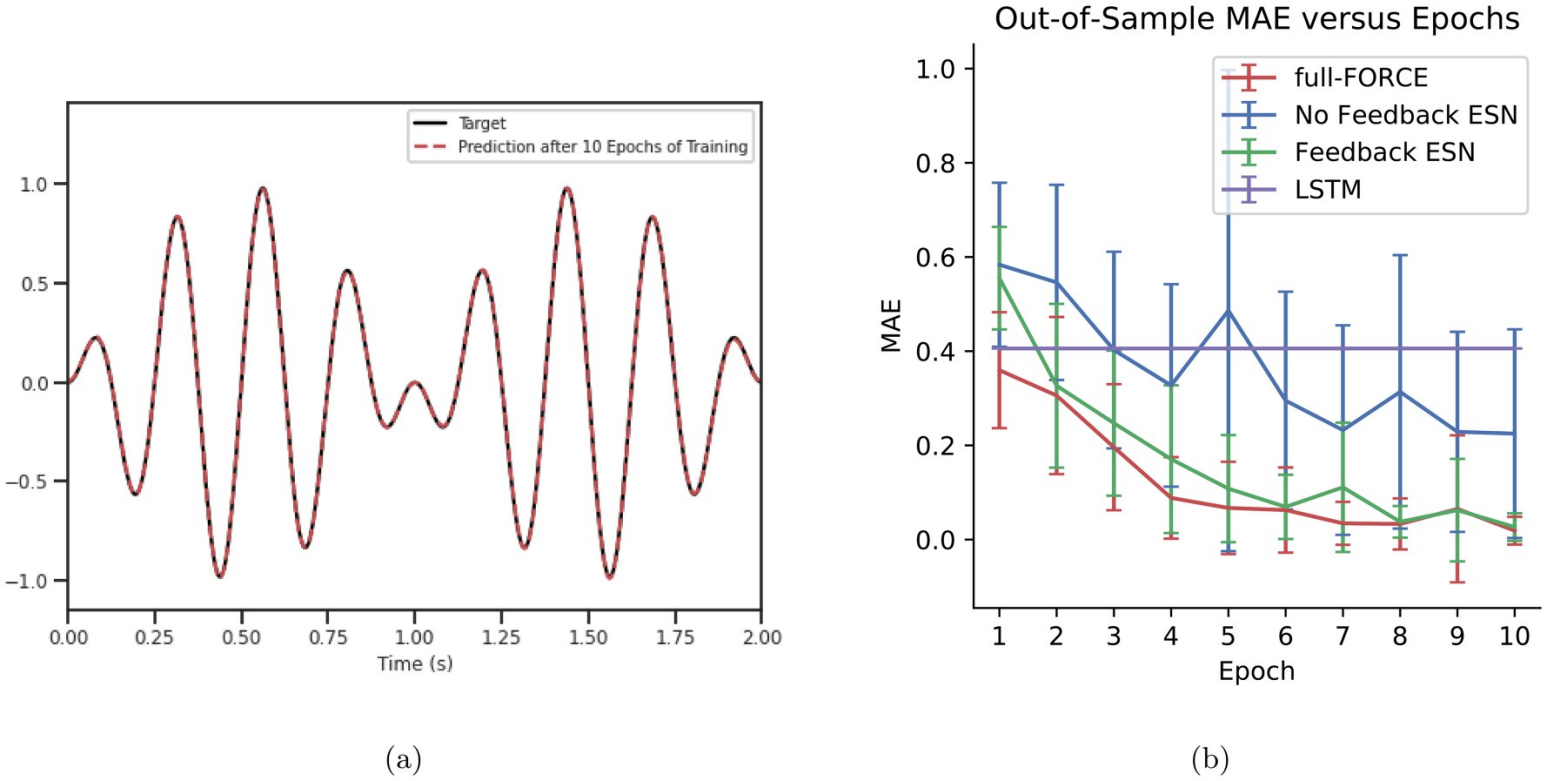
FORCE-training different network architectures to generate an autonomous periodic function. (A) Target output and full-FORCE output after 10 epochs using 400 fully connected neurons. (B) MAE comparison of different FORCELayers and standard LSTM trained by BPTT with ADAM.

Fig 4 illustrates experiments performed on a target made up of a sum of sine waves from [15]. The input is constant for the first 20 time steps (out of 801) and silent for the remaining time steps. A dummy hint input of zeroes is used for full-FORCE models and as an additional input dimension for FORCE models. For Fig 4B, the same input and target is passed into the model during training as validation data to be evaluated at the end of each epoch of training. The validation mean absolute error (MAE) is treated as out-of-sample error in Fig 4B. Each FORCE variant trained network had 400 fully connected neurons and trained for 10 epochs, with each experiment repeated 10 times with different randomly initialized weights to obtain the standard deviation / error bars.

The FullFORCEModel trained NoFeedbackESN generally achieved equal or superior out of sample performance compared to FORCEModel trained EchoStateNetwork and NoFeedbackESN after 10 epochs of training. Unlike the FORCE variant trained networks, decreasing out of sample MAE over the first 10 epochs was not observed for an equivalent Keras LSTM network trained using BPTT with ADAM. This demonstrates the limitations of gradient-based methods in learning dynamical targets in the absence of inputs over a large number of time steps and with limited training data.

**Fig 5:**
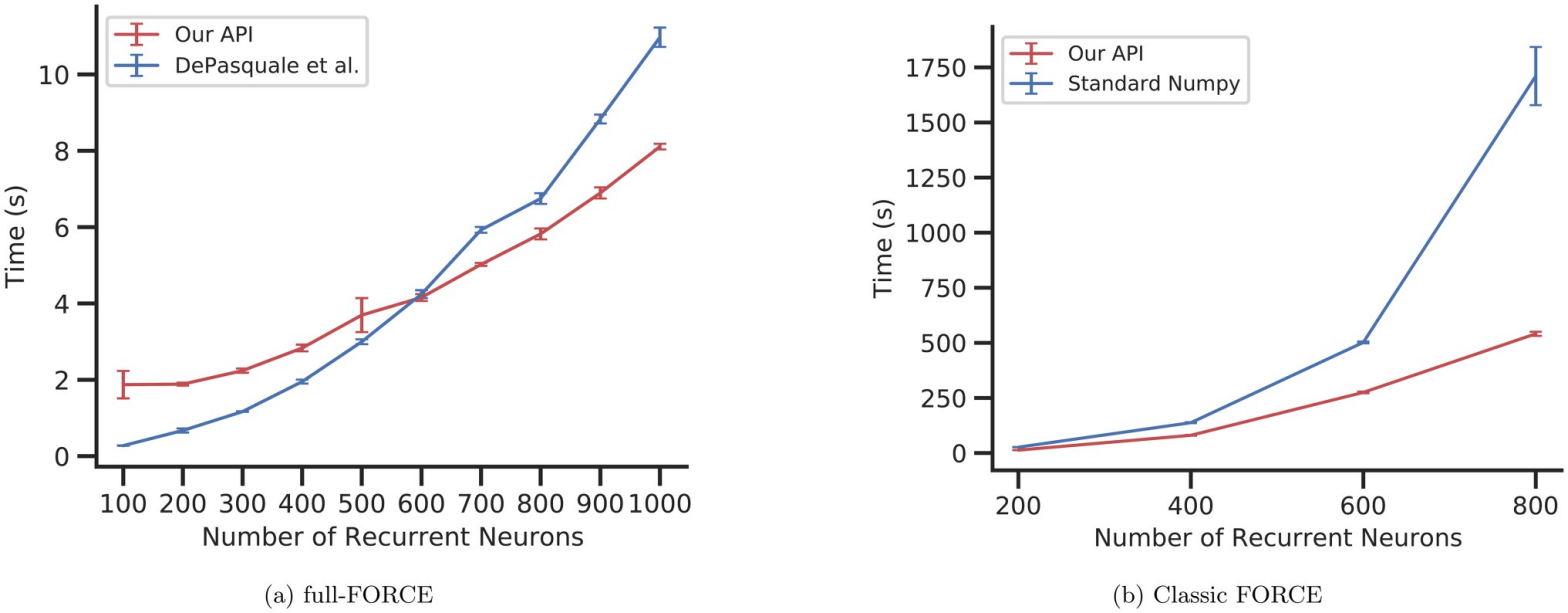
Runtime comparison using our API vs published/standard Python implementations. (A) full-FORCE (average across 10 epochs; error bars: standard deviation across 10 random initializations) (B) classic FORCE (average across 5 epochs; error bars: standard deviation across 5 random initializations).

We next compare the runtime of our implementation to reference implementations in standard Python/Numpy [28]. Experiments in Fig 5 were performed on Google Colab (Dual Core Intel(R) Xeon(R) CPU @ 2.20GHz CPU). For all experiments in Fig 5, recurrent weights are updated by the indicated algorithm while output weight are updated using FORCE, which has a time complexity of *O*(*N*^2^) (due to *P* matrix update) for a scalar target. FORCE and full-FORCE recurrent weight updates have a time complexity of *O*(*N*^3^) and *O*(*N*^2^) respectively (see Section 2.1 and Section A.3 in S1 Appendix).

Fig 5A compares the running time of our implementation of full-FORCE against the implementation from [15] on the ESN architecture without feedback. Networks with 100 to 1000 neurons were trained over 10 epochs using the same input, dummy hint, and target as Fig 4, with the mean running time across the 10 epochs being recorded. Each experiment was repeated 10 times to obtain the error bars. Fig 5A shows that the full-FORCE implementation in our API has a lower running time compared to the implementation by [15] in networks with >600 neurons.

Fig 5B compares the running time of our API with a standard Numpy implementation of FORCE in Python on an echo state network without feedback. The same input and target as Fig 5A, downsampled by a factor of two to run faster (resulting in 401 time steps), was used. Networks with 200 to 800 neurons were trained over 5 epochs with the mean running time across the 5 epochs being recorded. Each experiment was repeated 5 times to obtain the error bars. Fig 5B shows that our API has a faster running time for all network sizes.

For discussion of using the Accelerated Linear Algebra (XLA) library to further improve performance, see Section A.8 in S1 Appendix.

### 3.2 Learning the Lorenz attractor with spiking RNNs

**Fig 6:**
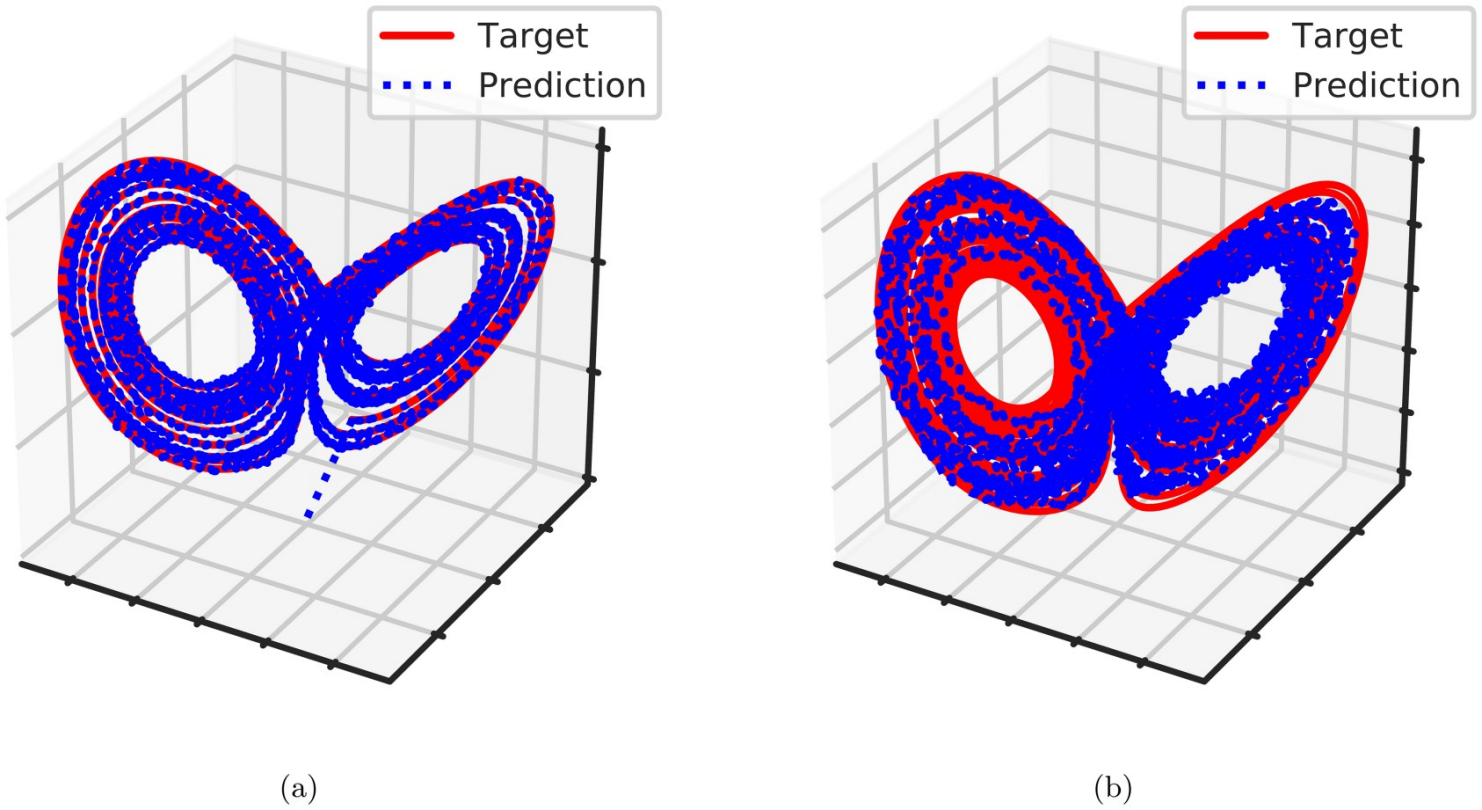
Trained Lorenz attractor dynamics in spiking networks. (A) Network output during training with FORCE updates every 50 time steps for *T* between 5 and 25 seconds. (B) Network output during inference (*T* > 25*s*).

Next, we show how our API can be used to FORCE-train a spiking neural network to output complex autonomous dynamics (Fig 6) [16]. The experiment ran for *T* = 50*s* with *dt* = 0.00001*s* using a network of Theta spiking neurons (see Section A.2 in S1 Appendix) and a modified SpikingNNModel. A network of 5000 Theta neurons with a sparsity level of 0.1 was first run through only the forward pass without FORCE weight updates for the first 5*s*. Then FORCE updates was applied every 50 time steps for the next 20*s*, then turned off for the remaining 25*s*. We find that the network faithfully outputs the target during training (Fig 6A) and remains close to the target after the ground truth signal is taken away (Fig 6B).

### 3.3 Learning a delayed response task with spiking RNNs

**Fig 7:**
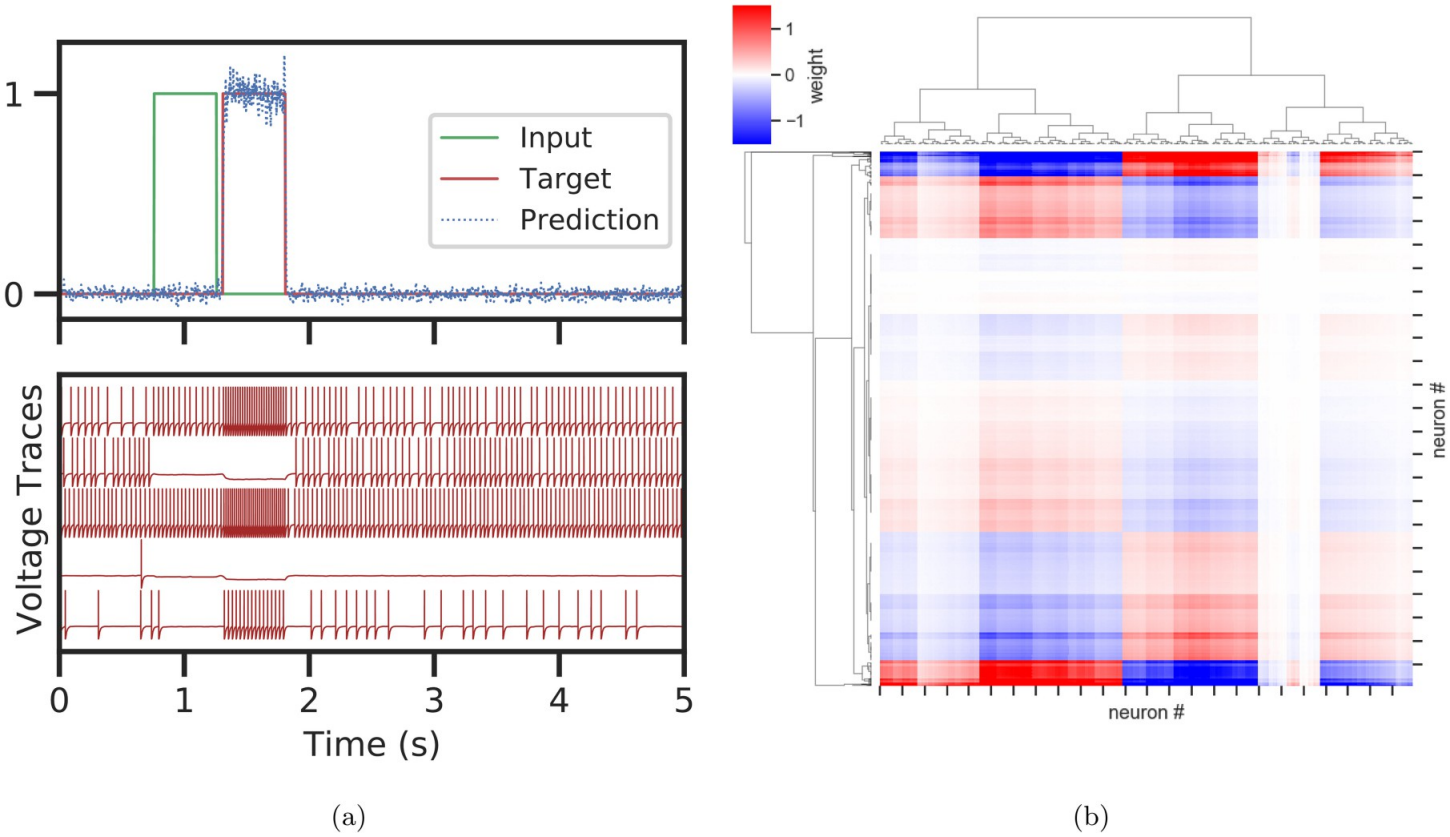
Delayed response task in a network of 5000 spiking leaky-integrate-and-fire neurons. (A) Top: Model inference performed on a randomly generated input and target. Bottom: Voltage traces for 5 neurons during inference. (B) Clustered effective weight matrix (downsampled in each axis by a factor of 5) shows block structure.

Next, we show how our API can be used to FORCE-train a network of spiking leaky-integrate-and-fire (LIF) neurons to perform a delayed response task (Fig 7). The input is a boxcar function that is on for 0.5 s. The network must wait until the input returns to zero, plus a fixed delay of 50 ms, before outputting the same input boxcar.

For training, 10 delayed response input-output examples were randomly generated, each lasting for *T* = 5*s* with time step *dt* = 0.0001*s*. A LIF network with 5000 fully connected neurons is trained using a custom SpikingNNModel for 5 epochs on the 10 examples with FORCE weight updates applied every 50 time steps. For inference, another input-output with delay example with the same parameters is randomly generated, passed through the model, and the results are shown in Fig 7A.

### 3.4 Modeling neural dynamics and learning effective weights

**Fig 8:**
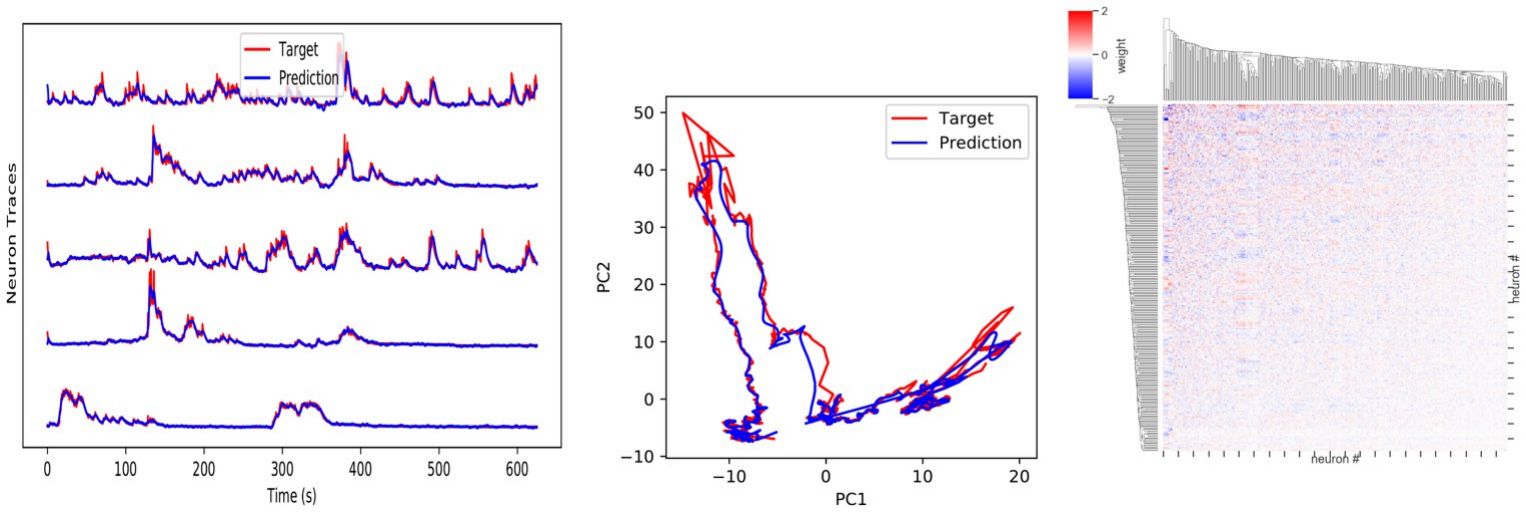
Reproducing neural dynamics recorded from larval zebrafish subpallium. (A) Target and network output for 5 example neurons after training ends. (B) Target and predicted trajectories of the trained network projected onto the first 2 PCs. (C) Learned effective weight matrix.

Constraining theoretical models with data is a central goal in neuroscience research. We show how our API can be used to learn a hypothesis for the effective network weights that generated observed neural data, following [22] (Fig 8). We drew from a dataset of single-cell resolution whole brain-area recordings from living larval zebrafish [22]. To model a full brain area, we initialized a fully recurrently connected network (excluding self-loops) with model neurons in one-to-one correspondence with neurons in the subpallium of a larval zebrafish brain (*N* = 365). In our dataset, each neuron in this brain area was recorded for 2500 time steps (*dt* = 0.25*s*), which were used as the target for training a ConstrainedNoFeedbackESN network with BioFORCEModel. The input and target are also passed in as the validation data to enable early stopping based on validation error. After training ends, the input / target is passed through for one additional epoch without FORCE updates to generate Fig 8. We find that our trained network closely reproduces the recorded trajectories; we can then analyze the learned effective weight matrix as a proxy for the unobservable neural weight matrix.

## Availability and future directions

RNNs have revolutionized the modeling of sequential data, but credit assignment across long time-horizons continues to pose a challenge [7, 8]. RLS-type training rules enjoyed early success with RNNs [25], but have been superseded by gradient-based algorithms. One RLS-type rule, the FORCE algorithm, has made a resurgence in the field of neuroscience as a way to accomplish computation using the internal chaotic dynamics of an RNN directly, drawing inspiration from recurrently connected brain regions which solve temporal tasks in a similar way [12, 15, 16, 29]. We provide a TensorFlow-compatible software library for FORCE-training RNNs, and validate our method in experiments in rate and spiking RNNs as well as with real neural data. This design allows our models to easily be incorporated into an existing TensorFlow training and evaluation workflow.

Our library currently supports FORCE learning with RLS as well as basic classic reservoir methods. In our basic dynamics simulations, equivalent loss was achieved in far fewer epochs with RLS compared to BPTT, though it is known that reservoir methods fail to generalize as well as gradient methods when presented with reduced-order observations [11]. Recent work has outlined one interpretation of an RLS rule as approximating a gradient with an adaptive learning rate [9]; future work could further explore this connection to better integrate these two fields. Advances in algorithms for reservoir computing may also further improve both training time and test performance [30]. We plan to continue expanding the tension package with additional models, tasks, and alternative training paradigms, hopefully with contributions from the computational neuroscience community. We hope our work will provide a common starting point for FORCE users to develop models and perform experiments, enabling faster iteration and lowering barriers to entry in training chaotic networks.

## Supporting information

S1 Appendix Additional information regarding algorithms implemented in our package and theory, usage and performance optimization, and supplementary discussion of related work. (PDF)

S1 Fig Runtime comparison of training a network with classic FORCE using our API without and with XLA optimization on CPU. For low neuron number performance is comparable, but XLA reduces training time for larger numbers of neurons by a factor of up to 3x.
(TIF)
