# Supplementary material for: tension: A Python package for FORCE learning
Source: PLoS Comput Biol. 2022 Dec 19;18(12):e1010722. doi: 10.1371/journal.pcbi.1010722 (PMC9810194; doi:10.1371/journal.pcbi.1010722)
Supplement: S1 Appendix — (PDF) [file pcbi.1010722.s001.pdf]

# A Appendix

## A.1 Classic FORCE update equations

Below are the equations for implementing classic FORCE with trainable output weights from (1). Similar equations for FORCE with trainable recurrent weights (1), full-FORCE (2), and FORCE in spiking networks (3) can be found in the associated publications.

$\mathbf{x}^T(t) \leftarrow \mathbf{x}^T(t-\Delta t) + \Delta t \dot{\mathbf{x}}^T(t)$ ; /* update $\mathbf{x}, \mathbf{r}$ using Eqn ?? */

$\mathbf{r}^T(t) \leftarrow H(\mathbf{x}^T(t))$ ; /* $H(.)$ is an activation func. */

$\mathbf{z}^T(t) \leftarrow \mathbf{r}^T(t)\mathbf{w}_{out}(t-\Delta t)$ ; /* calculate output */

$\mathbf{P}(t) \leftarrow \mathbf{P}(t-\Delta t) - \frac{\mathbf{P}(t-\Delta t)\mathbf{r}(t)\mathbf{r}^T(t)\mathbf{P}(t-\Delta t)}{1+\mathbf{r}^T(t)\mathbf{P}(t-\Delta t)\mathbf{r}(t)}$ ; /* update P */

$\mathbf{e}_-(t) = \mathbf{z}(t) - \mathbf{f}_{out}(t)$; /* calculate error */

$\Delta \mathbf{w}_{out} \leftarrow \mathbf{P}(t)\mathbf{r}(t)\mathbf{e}_-^T(t)$ ; /* calculate pseudogradient */

$\mathbf{w}_{out}(t) \leftarrow \mathbf{w}_{out}(t-\Delta t) - \Delta \mathbf{w}_{out}$ ; /* update weights */

## A.2 Spiking neuron models

The continuous time voltage rate equations of the Theta, LIF, and Izhikevich spiking neural networks as outlined in (3) are summarized below. The voltage for the Theta neuron is governed by:

$$\dot{\boldsymbol{\theta}} = (1 - \cos(\boldsymbol{\theta}(t))) + \pi^2 \mathbf{I}(t)(1 + \cos(\boldsymbol{\theta}(t)))$$

The voltage for the LIF neuron is governed by:

$$\tau_m \dot{\mathbf{v}} = -\mathbf{v}(t) + \mathbf{I}(t)$$

The voltage for the Izhikevich neuron is governed by:

$$\begin{aligned} C\dot{\mathbf{v}} =& k(\mathbf{v}(t) - v_r)(\mathbf{v}(t) - v_t) - \mathbf{u}(t) + \mathbf{I}(t) \\ \dot{\mathbf{u}} =& a(b(\mathbf{v}(t) - v_r) - \mathbf{u}(t)) \end{aligned}$$

where $\mathbf{I}(t) = I_{Bias} + \mathbf{s}(t)$.

## A.3 full-FORCE algorithm

full-FORCE (2) is a modified architecture for FORCE training in which a target-generating network is used to derive target hidden activations for each neuron in the primary network to more efficiently update $\mathbf{w}_{\mathbf{R}}(t)$. The discrete time forward pass of this target generating network is (denoted by subscript $D$):

$$\tau \frac{\Delta \mathbf{x}_D^T}{\Delta t} = -\mathbf{x}_D^T(t-\Delta t) + \mathbf{r}_D^T(t-\Delta t)\mathbf{w}_{R,D} + \mathbf{f}_{in}^T(t)\mathbf{w}_{in,D} + \mathbf{f}_{hint}^T(t)\mathbf{w}_{hint,D} + \mathbf{f}_{out}^T(t)\mathbf{w}_{F,D}$$

where all weights of the target generating network are randomly initialized and not trainable. The recurrent weights of the primary network are updated using:

$$\begin{aligned} \mathbf{e}_{FF}^T(t) &= \mathbf{r}^T(t)\mathbf{w}_R(t-\Delta t) - \mathbf{r}_D^T(t)\mathbf{w}_{R,D} - \mathbf{f}_{hint}^T(t)\mathbf{w}_{hint,D} - \mathbf{f}_{out}^T(t)\mathbf{w}_{F,D} \\ \mathbf{w}_R(t) &= \mathbf{w}_R(t-\Delta t) - \mathbf{P}(t)\mathbf{r}(t)\mathbf{e}_{FF}^T(t) \end{aligned}$$

## A.4 FORCE-training spiking RNNs

Spiking neural networks have attracted significant recent interest both in the neuroscience and the machine learning community (4; 5; 6). A FORCE approach can also be used to train spiking RNNs by incorporating synaptic filtering dynamics, which translate discrete presynaptic spikes into continuous postsynaptic currents. Based on (3), a simple discrete time single exponential synaptic filter is:

$$\frac{\Delta \mathbf{r}}{\Delta t} = -\frac{\mathbf{r}(t-\Delta t)}{\tau_s} + \frac{1}{\tau_s} \sum_{t_{jk}<t-\Delta t} \delta(t-\Delta t - t_{jk})$$

and the discrete time double exponential synaptic filter is:

$$\begin{aligned}\frac{\Delta \mathbf{r}}{\Delta t} &= -\frac{\mathbf{r}(t-\Delta t)}{\tau_d} + \mathbf{h}(t-\Delta t)\\ \frac{\Delta \mathbf{h}}{\Delta t} &= -\frac{\mathbf{h}(t-\Delta t)}{\tau_r} + \frac{1}{\tau_r\tau_d} \sum_{t_{jk}<t-\Delta t} \delta(t-\Delta t - t_{jk})\end{aligned}$$

where $t_{jk}$ indicates the time of the $k$th spike for the $j$th neuron. We include specific spiking neuron models in Section A.2.

## A.5 Spiking neural networks

Per (3), the continuous time single exponential synaptic filter is given by:

$$\dot{\mathbf{r}} = -\frac{\mathbf{r}(t)}{\tau_s} + \frac{1}{\tau_s} \sum_{t_{jk}<t} \delta(t - t_{jk})$$

and the double exponential synaptic filter is given by:

$$\begin{aligned}\dot{\mathbf{r}} &= -\frac{\mathbf{r}(t)}{\tau_d} + \mathbf{h}(t)\\ \dot{\mathbf{h}} &= -\frac{\mathbf{h}(t)}{\tau_r} + \frac{1}{\tau_r\tau_d} \sum_{t_{jk}<t} \delta(t - t_{jk})\end{aligned}$$

where $t_{jk}$ indicates the time of the $k$th spike for the $j$th neuron.

## A.6 Connection to Echo-State Networks

Like FORCE, classic ESN training(7) harnesses chaotic internal dynamics within an RNN to perform time-series prediction tasks. The main difference between ESN learning and FORCE learning is that in the ESN, the target function $f(t)$ is used as the feedback signal during training, while in FORCE, the actual network output $z(t)$ is used as the feedback signal, which empirically improves stability (1).

## A.7 Connection to gradient methods

The shared goal of ESNs and FORCE is to train RNNs conscious of and explicitly manipulating dynamics internally generated within the RNN reservoir, with the hope of expressing long-term dependencies in a basis of these dynamics (1; 8). This represents a distinct conceptual approach from gradient methods such as backpropagation through time (BPTT), which aim to gradually reduce training error using small, full-rank (in general) updates to the trainable weights proportional to the gradient of the error

function. In the chaotic regime of an RNN, gradient methods may fail to converge if the gradient degenerates (8). In contrast, RNNs with initial chaotic spontaneous activity converge *more* quickly and find more robust solutions under FORCE learning (1).

An alternative perspective considers whether the RLS delta update can be (approximately) written as the true gradient of some objective function. (9) address a similar problem: given the linear least-squares loss function

$$J(\mathbf{W}) = \sum_{i=1}^{S} \sum_{t=t_0}^{T} \lambda^{s-i} ||\mathbf{Z}_{i,t} - \mathbf{Z}_{i,t}||_F^2$$

with network parameters $\mathbf{W}$, $S$ samples with $T$ timepoints and linear readouts $\mathbf{Z}$, the authors show that an RLS update roughly approximates an SGD update with an adaptive learning rate.

In our work here, as in the majority of existing work using FORCE, the gradient of the error in the output is not used. The above-mentioned approximation notwithstanding, the update computed by FORCE is not in general proportional to the gradient of the error. Nonetheless, interpolating between FORCE and gradient-based weight updates represents a potentially promising avenue for future work.

## A.8 Improving performance using XLA

The performance of FORCE learning is largely dictated by the speed of the underlying linear algebra library used to compute the matrix operations core to RLS. An advantage of using a TensorFlow-based framework is that the Accelerated Linear Algebra (XLA) optimizing compiler can be deployed easily to speed up the code with no code changes required (10). **??** illustrates the running time of training an `NoFeedbackESN` using `FORCEModel` without and with XLA optimization. Experiments were performed on a 32-core AMD EPYC 7551P processor @ 2 GHz. The same input and target as Fig **??** was used. Networks were trained for 5 epochs with the average running time being recorded. The error bars were obtained by running each experiment 5 times. The performance improvement afforded by utilizing XLA may vary based on specific hardware used; due to known compatibility issues we were unable to perform this experiment on GPU.

# References


1. Sussillo D, Abbott LF. Generating coherent patterns of activity from chaotic neural networks. Neuron. 2009;63(4):544-57.

2. DePasquale B, Cueva CJ, Rajan K, Escola GS, Abbott L. full-FORCE: A target-based method for training recurrent networks. PloS one. 2018;13(2):e0191527.

3. Nicola W, Clopath C. Supervised learning in spiking neural networks with FORCE training. Nature communications. 2017;8(1):1-15.

4. Huh D, Sejnowski TJ. Gradient descent for spiking neural networks. Advances in neural information processing systems. 2018;31.

5. Tavanaei A, Ghodrati M, Kheradpisheh SR, Masquelier T, Maida A. Deep learning in spiking neural networks. Neural networks. 2019;111:47-63.

6. Pfeiffer M, Pfeil T. Deep learning with spiking neurons: opportunities and challenges. Frontiers in neuroscience. 2018:774.

7. Jaeger H, Haas H. Harnessing nonlinearity: Predicting chaotic systems and saving energy in wireless communication. science. 2004.

8. Lukoševičius M, Jaeger H. Reservoir computing approaches to recurrent neural network training. Computer Science Review. 2009;3(3):127-49.

9. Zhang C, Song Q, Zhou H, Ou Y, Deng H, Yang LT. Revisiting Recursive Least Squares for Training Deep Neural Networks. arXiv; 2021. Available from: `https://arxiv.org/abs/2109.03220`.

10. Sabne A. XLA : Compiling Machine Learning for Peak Performance; 2020.
