# Supplementary figures and images for: tension: A Python package for FORCE learning
Source: PLoS Comput Biol. 2022 Dec 19;18(12):e1010722. doi: 10.1371/journal.pcbi.1010722 (PMC9810194; doi:10.1371/journal.pcbi.1010722)

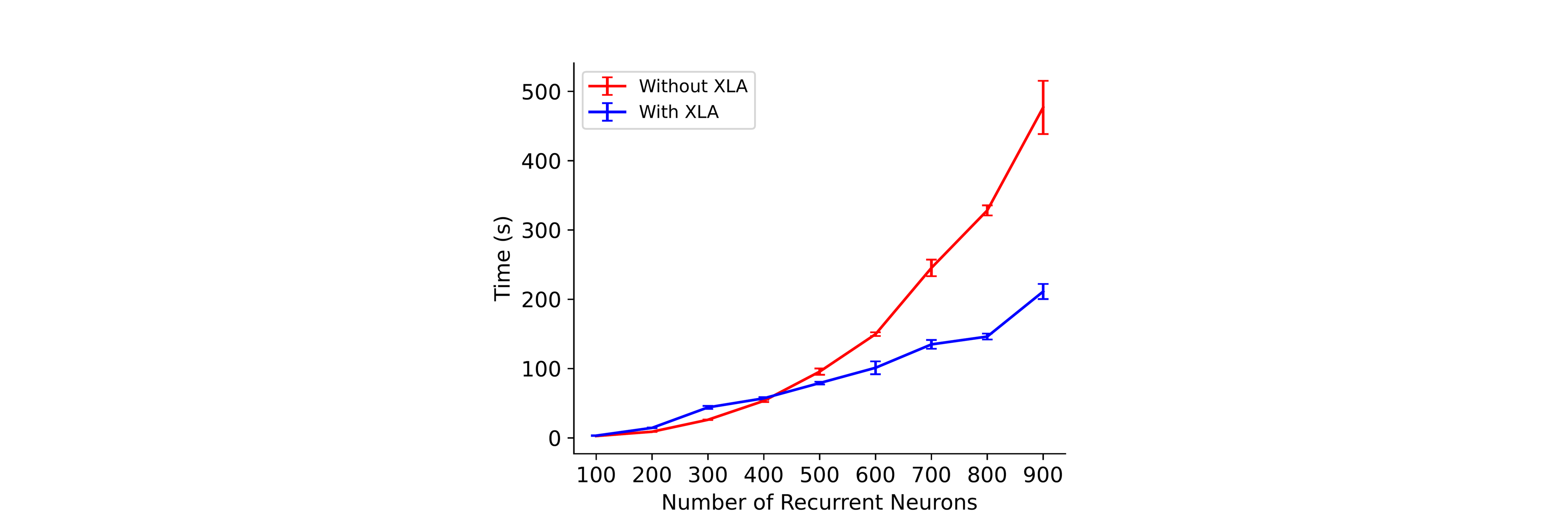

Supplement: S1 Fig — For low neuron number performance is comparable, but XLA reduces training time for larger numbers of neurons by a factor of up to 3x. (TIF) [file pcbi.1010722.s002.tif]
